# A systematic review of observational methods used to quantify personal protective behaviours among members of the public during the COVID-19 pandemic, and the concordance between observational and self-report measures in infectious disease health protection

**DOI:** 10.1186/s12889-022-13819-0

**Published:** 2022-07-28

**Authors:** Rachel Davies, Fiona Mowbray, Alex F. Martin, Louise E. Smith, G. James Rubin

**Affiliations:** National Institute of Health Research Health Protection Research Unit in Emergency Preparedness and Response at King’s College London, in partnership with the UK Health Security Agency, London, UK

**Keywords:** COVID-19, Hand Washing, Face Mask, Social Distancing, Behavioural Adherence, Observational

## Abstract

**Objectives:**

To assess the quantity and quality of studies using an observational measure of behaviour during the COVID-19 pandemic, and to narratively describe the association between self-report and observational data for behaviours relevant to controlling an infectious disease outbreak.

**Design:**

Systematic review and narrative synthesis of observational studies.

**Data sources:**

We searched Medline, Embase, PsychInfo, Publons, Scopus and the UK Health Security Agency behavioural science LitRep database from inception to 17th September 2021 for relevant studies.

**Study selection:**

We included studies which collected observational data of at least one of three health protective behaviours (hand hygiene, face covering use and maintaining physical distance from others (‘social distancing’) during the COVID-19 pandemic. Studies where observational data were compared to self-report data in relation to any infectious disease were also included.

**Data extraction and synthesis:**

We evaluated the quality of studies using the NIH quality assessment scale for observational studies, extracted data on sample size, setting and adherence to health protective behaviours, and synthesized results narratively.

**Results:**

Of 27,279 published papers on COVID-19 relevant health protective behaviours that included one or more terms relating to hand hygiene, face covering and social distancing, we identified 48 studies that included an objective observational measure. Of these, 35 assessed face covering use, 17 assessed hand hygiene behaviour and seven assessed physical distancing. The general quality of these studies was good. When expanding the search to all infectious diseases, we included 21 studies that compared observational versus self-report data. These almost exclusively studied hand hygiene. The difference in outcomes was striking, with self-report over-estimating observed adherence by up to a factor of five in some settings. In only four papers did self-report match observational data in any domains.

**Conclusions:**

Despite their importance in controlling the pandemic, we found remarkably few studies assessing protective behaviours by observation, rather than self-report, though these studies tended to be of reasonably good quality. Observed adherence tends to be substantially lower than estimates obtained via self-report. Accurate assessment of levels of personal protective behaviour, and evaluation of interventions to increase this, would benefit from the use of observational methods.

**Supplementary Information:**

The online version contains supplementary material available at 10.1186/s12889-022-13819-0.

## Background

Throughout the COVID-19 pandemic, members of the public have been urged to engage in a set of behaviours intended to reduce transmission of the SARS-CoV-2 virus. These have included recommendations to practice frequent hand hygiene, avoid close contact with other people (‘social distancing’) and to wear a face covering to prevent spread through respiratory droplets [1, 2]. Although these interventions have been shown to be effective in reducing the transmission of SARS-CoV-2 (https://www.bmj.com/content/375/bmj-2021-068302), none of the interventions will work if people do not adhere to them or understand the messaging on when they should adhere [3].

To date, public engagement with recommended behaviours has primarily been monitored by behavioural scientists, public health agencies and national governments though the use of self-report questionnaires. Using self-report methods to collect data has many benefits. Self-reported data can be quick, easy and relatively inexpensive to obtain from large numbers of participants. The association between self-reported behaviour and other self-reported variables is also relatively straightforward to examine. For many outcomes, self-report can be a good proxy measure for actual behaviour [4]. For example, self-report can be a useful way to assess whether someone has been vaccinated or not [5]. For other behaviours, self-report may be less valid [6]. This may be particularly true for frequently performed behaviours that are difficult to remember (e.g. frequency of handwashing in the past 24 hours) or that would be socially undesirable to admit (e.g. breaking legally enforceable rules around self-isolation) [7, 8, 9].

In the context of COVID-19, regularly collected measures of behaviour that do not rely on self-report are rare. Notable exceptions include: mobility data based on mobile phone locations [10]; footfall data in city centres [11]; and official statistics on vaccine uptake based on electronic records [12]. Most of these examples relate to where people are located or whether they engage with health services.

There are fewer regularly collected data based on direct observation quantifying whether and how people engage with COVID-19 protective behaviours. To assist public health agencies in considering whether to collect more observational data, we conducted a systematic review of the use of observational measures of COVID-19 relevant behaviours. We focussed on studies that directly observed the performance of protective behaviours, excluding measures of mobility or location. Our aims were to assess: 1) the quantity of observational studies conducted during COVID-19; 2) the quality of these studies; and 3) the association between self-report and observational data. While we only included COVID-19 related studies for aims one and two, given a lack of data found during screening, we expanded our inclusion criteria for aim three and included studies relating to any infectious disease outbreak.

## Methods

### Protocol and registration

This review follows the PRISMA framework and is registered with PROSPERO registration number CRD42021261360. The study protocol is available from: https://www.crd.york.ac.uk/prospero/display_record.php?ID=CRD42021261360.

### Search strategies

For aims one and two, we searched the following electronic databases from inception to 17th September 2021: Medline, Embase, PsycInfo, Publons, Scopus, and the UK Health Security Agency behavioural science COVID-19 Literature Repository database (BSIU LitRep Database. Google Docs. Available from: https://docs.google.com/spreadsheets/d/1qfR4NgnD5hTAS8KriPaXYhLu1s7fpZJDq8EIXQY0ZEs/edit#gid=369408275 (Accessed November 2021)). Databases were searched for articles containing MeSH terms or keywords relating to COVID-19 (e.g. “SARS-CoV-2”, “novel coronavirus”), hand hygiene, physical distancing, or face coverings (e.g. “hand washing”, “face mask”, “physical distancing”) and an observational method (e.g. “observational study”, “videorecording”). Full details of our search strategies are available in Supplementary material.

For aim three, we searched Medline, Embase and PsycInfo from inception to 17th September 2021. These were searched for articles containing MeSH terms or keywords relating to hand hygiene, physical distancing, face covering and direct observation. We did not include specific search terms for infectious disease as this was already the focus of the majority of papers investigating the three relevant behaviours.

For both search strategies we examined the reference sections of any pertinent studies and reviews for further references.

### Eligibility criteria

For aims one and two, we included studies that were published in English since January 2020, contained an observational measure of hand hygiene, physical distancing or face covering use in relation to COVID-19, assessed these behaviours among the general public or healthcare workers, and contained original data. We excluded studies that contained only location-based data, for example, mobile phone data that measured where in space people were located (rather than what they were doing). We also excluded the use of used crowd density measurements where physical distancing of individuals within the crowd could not be determined. Studies were also excluded if they recorded impressionistic perceptions of behaviour rather than using a systematic method such as using unsystematic sampling methods or retrospective methods based on recall of behaviours.

For aim three, we included studies published in English (no date restrictions), that related to infectious disease control for any pathogen and that contained an observational measure of one or more of our defined behaviours compared to a self-report measure. We excluded studies that contained only self-report or observational data.

### Study selection

Titles and abstracts were independently double screened by two separate reviewers (RD screened all citations, FM screened half the citations and AFM screened the other half) using Sysrev Software to identify potentially eligible studies and record decisions. Full texts were then independently double screened (RD screened all citations, FM screened half the citations and AFM screened the other half), with any uncertainties resolved through discussion.

### Data extraction, items and risk of bias

Two reviewers (RD, FM) extracted data from included studies. Study and participant characteristics were noted, including study design, sample size, number of opportunities for specified behaviours, location of observation, population characteristics and prevalence of adherence. Where needed, further details were sought by contacting study authors. For aims one and two, where papers contained a pre and post COVID-19 data collection period, only data collected during the COVID-19 pandemic were included in the narrative synthesis.

Studies were assessed for quality using the National Institutes of Health (NIH) Quality Assessment Tool for Observational Cohort and Cross Sectional Studies [13]. Where disagreements were identified, the relevant reviewers discussed the relevant sections of the paper to check if they had misinterpreted any element. Where needed, a third reviewer was asked for their advice and / or the relevant table entry for a study was adjusted to account for any ambiguity.

## Results

Twenty-seven thousand two hundred seventy-nine published papers were identified that included terms relating to COVID-19, and one or more terms relating to hand hygiene, face covering and social distancing. When the term ‘observational’ and related terms were added, 2589 papers were identified and these were screened for aims one and two, from which 105 were selected as potentially relevant to the review. Of these, 57 were excluded. A total of 48 studies met the inclusion criteria (Fig. S1). For aim three we screened 3331 papers, from which 133 were deemed potentially relevant following abstract screening. Of these, 21 were included in the review (Fig. S2).

### Aim one: quantity of studies using observational measures

We included 48 [14–, 15, 16, 17, 18, 19, 20, 21, 22, 23, 24, 25, 26, 27, 28, 29, 30, 31, 32, 33, 34, 35, 36, 37, 38, 39, 40, 41, 42, 43, 44, 45, 46, 47, 48, 49, 50, 51, 52, 53, 54, 55, 56, 57, 58, 59, 60, 61] studies containing an observation component during the COVID-19 pandemic. In total these included at least 116,169 participants and at least 36,060,422 behavioural observational events.

Of the included studies, 39 used direct observers, one used an automated measurement to assess hand hygiene, five used video observations and three used mixed methods including; observation supplemented with a survey, observation supplemented with media data and in-person observation plus automated technology.

Of the 48 included studies, 35 looked at wearing a face covering (five in healthcare workers, 30 in the general public), 17 studies looking at hand hygiene (12 in healthcare workers, five in the general public), and seven looked at physical distancing (one in healthcare workers and six in the general public).

Six studies contained an interventional component intended to improve adherence.

Studies had been conducted in Asia (*n* = 18), North America (*n* = 15), Europe (14), Africa (*n* = 2), and Australia (*n* = 1).

The most common setting for observation was in hospitals (*n* = 20), in stores or shopping centres (*n* = 12), on public streets (*n* = 11), on public transport (*n* = 7), in parks (*n* = 4), high schools or universities (*n* = 3), community healthcare (*n* = 2) and residential care homes (*n* = 1).

For papers for which it could be determined (*N* = 43), sample size varied between 41 and 17,200 (median = 780).

The median number of behavioural observation events for each study was 1020, with a minimum of 41 and maximum of 35,362,136. Two studies [55, 56] had a very high number of observations, one with 35,362,136 opportunities and one with 593,118.

Characteristics of all included studies are available in Tables S1 and S3.

### Aim two: quality of studies using observational measures

**Fig. 1:**
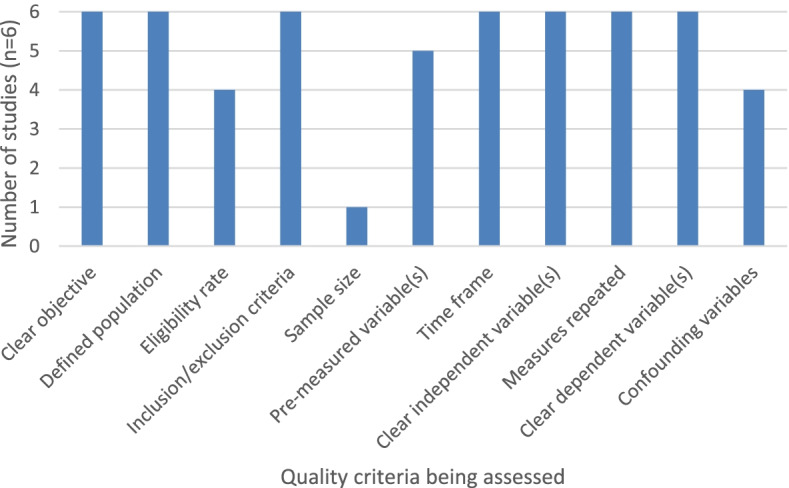
Number of intervention studies displaying relevant aspects of NIH quality assessment tool

**Fig. 2:**
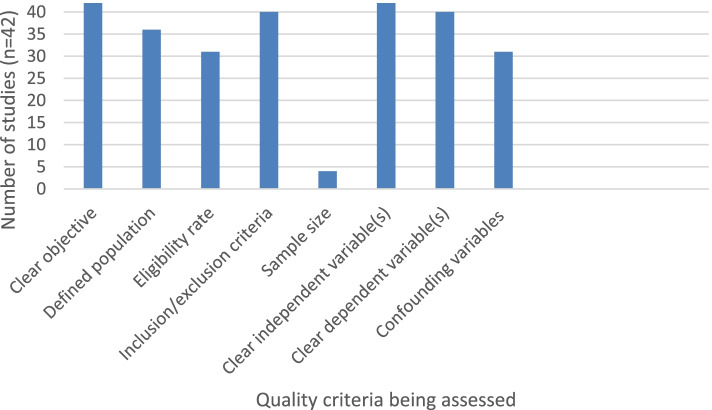
Number of non-intervention studies displaying relevant aspects of NIH quality assessment tool

Studies with interventions intended to improve adherence to protective behaviours (*n* = 6) were rated out of 11 relevant criteria on the NIH quality assessment checklist and studies with no interventions (*n* = 42) were rated out of eight relevant criteria. Studies with an intervention had a median score of 10, with a range of 8-11 (Fig. 1). Studies without an intervention had a median score of 7, with a range of 4-8 (Fig. 2). Overall, studies in both groups generally had clearly defined study objectives, populations and variables, however very few studies reported any sample size or power estimates.

### Aim three: observational data vs self-report

In total, 21 studies contained both an observational and self-report component (Table 1). Characteristics of all included studies are available in Tables S1, S2, S3 and S4. Three studies investigated COVID-19, while 18 investigated other infectious diseases or infectious disease practice pre-COVID-19. Of the three studies investigating behaviour during COVID-19, all three studied healthcare workers (one in Germany, one in the US and one in Thailand) and one also studied the general public (In Thailand) [25, 51, 52]. All three looked at hand hygiene adherence; one also looked at face covering use.

**Table 1:** Summary of included studies which reported an observation and self-report measure of health protective behaviour

Study	Population; location	Observed prevalence	Self-report prevalence
Henry (1992) [62]	ED physicians (12 staff plus rotating residents), medical students, nursing staff, and ancillary personnel; hospital emergency department	Overall face covering wearing adherence: 1.2%	Overall face covering wearing adherence: 25.5%
Raymond (2001) [63]	Tattoo artists; Minneapolis and St. Paul, MN	Overall adherence was 71%	Overall adherence was 83%
Cohen (2002) [64]	Dermatologists; outpatient dermatology clinics in Israel	7 (38.5%) physicians washed hands only after contact with suspected infected material and 1 (7.7%) before each physical examination. Seven physicians (53.9%) washed their hands 1–5 times a day	19 (37.3%) washed their hands only following contact with material suspected of being contaminated, 18 (35.3%) prior to examining every patient, 14 (27.5%) wash hands 1–5 times a day
Moret (2004) [65]	Healthcare workers (physicians, nurses, and nurse assistants); French university hospital	Overall adherence was 74%	Overall adherence was 74%
Snow (2006) [66]	60 students enrolled in a certified nursing program; on day 1 and day 30 of the clinical rotation.	Day 1. Student hand hygiene:49.1%. Day 30. Student hand hygiene:52.3%.	Based on a scale of 10-100. 10 being lowest possible commitment to handwashing. On day 30 students reported 93.0 before patient contact, 95.5 after patient contact, 84.9 before donning gloves, and 95.1 after removal of gloves.
Alemayehu (2009) [67]	Third and fourth year students; U.S. medical school over one academic year at the beginning of every core rotation (medicine, surgery, primary care medicine, paediatrics, obstetrics/gynaecology, neurology, psychiatry)	Overall adherence: 87.9%	Overall adherence: 87.9%
Soyemi, C (2010) [68]	Doctors and nurses; three types of hospitals, i.e., public, security forces, and private, in Eastern Saudi Arabia.	Overall adherence for physicians: 27% Overall adherence for nurses: 29%	Likert scale. Physicians mean self-report was 10.19/15 based on 3 hand hygiene opportunities, and nurses was 13.65/15 based on 3 opportunities.
Jessee (2013) [69]	Staff on 3 medical/surgical units; a 600-bed academic medical centre (AMC) and a 110-bed community medical centre (CMC) in the South-eastern United States	Hand hygiene before glove application, was present in 14% of the CMC and 21% of the AMC staff Hand hygiene on room exit was 25 and 78% respectively.	Hand hygiene before glove application was present in 63% CMC and 50% of AMC staff. Hand hygiene on room exit 16 was 84 and 94% respectively.
Kim (2013) [70]	Doctors; healthcare settings.	Adherence at baseline: 49.7% Adherence in fourth quarter of 2012: 82.3%	Adherence at baseline: 82.9% Adherence in fourth quarter of 2012: 93.8%
Dalen (2013) [71]	Doctors (13%), nurses (70%), housekeeping staff (9%) and visitors of patients (9%); cancer hospital.	Overall hand hygiene adherence, nurses: 47% Overall hand hygiene, doctors: 51%	Overall hand hygiene adherence, nurses: 88% Overall hand hygiene adherence, doctors: 85%
Lakshmi (2015) [72]	Healthcare workers (predominantly nurses); ICU in an oncology, BMT and neurosurgical centre in South India.	Use of alcohol based hand rub 98.5%, 5 moments of hand hygiene 88.5%, 6 steps of hand hygiene 65%.	Use of alcohol based hand rub 98.5%, 5 moments of hand hygiene 88.5%, 6 steps of hand hygiene 92.5%
O’Donoghue (2016) [73]	Healthcare workers, 76 radiographers, 17 nurses, and nine healthcare assistants; a radiography unit.	Overall adherence: 29% Post-test adherence: 51%	‘I wash my hands after using the rest room’ was rated a median of 5/5 on a Likert scale, quartiles 4-5 before and after intervention.
Galiani (2016) [74]	General public; households and community settings in Peru	Handwashing with soap was observed in only 16% of the events that required it. 20% of faecal contact events, 25% of eating events, 6% of child feeding events, and 10% of food preparation events.	Less than 50% reported hand washing at times of faecal contact. 39% reported with toilet use, 34% cleaning up after children, 68% when cooking or with food preparation and 31% when feeding a child.
Keller (2018) [75]	ED staff, 100 nurses, 13 staff emergency physicians, 25 medical interns, and various other professions (e.g. maintenance and nursing assistants). Non-ED consulting physicians and surgeons regularly visiting the ED; in an Emergency Department of the University Hospital Zurich	Hand hygiene adherence during baseline: 56%. Hand hygiene adherence during intervention: 64%	Hand hygiene adherence during baseline: 4.12. Hand hygiene during intervention 4.03 (on a Likert scale 1-5, 5 being always)
Baloh (2019) [76]	Healthcare workers; 3 large academic US hospitals	Hand hygiene before gloving was performed 42% of the time	Hand hygiene before gloving was reported 88% of the time
Le (2019) [77]	Healthcare workers (physicians, nurses, care assistants, and student nurses); large central hospital in Vietnam	Physicians overall hand hygiene adherence:14.6%. Nurses overall hand hygiene adherence: 38.8%	Physicians overall hand hygiene adherence: 67.2%. Nurses overall hand hygiene adherence: 97.8%
Woodard (2019) [78]	Healthcare workers (nurses, physicians, technicians) and patient interactions; 750-bed tertiary care hospital in Baltimore, Maryland.	Overall hand hygiene adherence: 35% of all opportunities. Overall entry and exit hand hygiene adherence: 90%	81% of the sample estimated they miss performing hand hygiene when they realize it should be performed 10-20% of the time. An additional 11% reported missing hand hygiene 30-40% of the time. 4% estimated they missed performing hand hygiene 90-100% of the time.
Kelcikova (2019) [79]	Doctors and nurses; eight hospitals in two countries (five in Slovakia and three in the Czech Republic)	Overall adherence: 67.7%	Overall adherence: 74.0%
Derksen (2020) [50]	Healthcare workers (physicians, midwives, and nurses); two German obstetric hospitals during and after the onset of the COVID-19 pandemic.	After declaration of pandemic overall hand hygiene adherence: 95%. After body fluid exposure risk: 100%.	After declaration of pandemic overall hand hygiene adherence: M = 5.03, SD = 0.75 on a 6 point scale. After body fluid exposure risk: M = 5.66, SD = 0.67.
Dowding (2020) [51]	400 U.S. home care nurses; community care organization	Overall hand hygiene adherence: 45.6%.	Overall hand hygiene adherence: 99.4%
Skuntaniyom (2021) [24]	119 healthcare workers (mostly nurses and laboratory workers) and 100 general public (mostly administrative workers and professionals); Thailand in two inpatient hospitals providing COVID-19 testing and treatment.	100% of patients and 100% healthcare workers wore face covering correctly. 35.2% of patients and 40.0% of healthcare workers correctly cleaned all areas of both hands.	86% of patients and 95.8% of healthcare workers wore a face covering correctly. 67% of patients and 84.9% of healthcare workers reported adhering to hand hygiene.

Self-reported and observed hand hygiene behaviour differed, with self-reported rates being around twice that of observed rates. The biggest difference seen in hand hygiene rates was 99% self-reported and 46% observed in a study of healthcare workers engaging in community-based patient care activities in the US [51]. Observed adherence in this study varied by the activity as well as by the period during the COVID-19 pandemic when assessments were made.

Self-reported and observed face covering wearing both had high rates of adherence, with 86% self-reported adherence among patients and 95.8% among healthcare workers in one Bangkok hospital, compared to 100% adherence when observed in both groups [24].

Of 18 studies investigating other infectious diseases, most studies (*n* = 15) looked at hand hygiene in a healthcare worker population [64, 65, 66, 67, 68, 69, 70, 71, 72, 73–, 75, 76, 77, 78, 79], while two studied it in the general public [63, 74]. One studied face covering use in healthcare workers [62]. None assessed physical distancing. Studies were conducted in Asia (*n* = 7), North America (*n* = 8), Europe (*n* = 3) and South America (*n* = 1).

Self-reported hand hygiene behaviour was higher than observed data in most studies (*n* = 11). The greatest differences were 31% self-reported versus 6% observed hand hygiene in the general public in Peru [79], and 67% self-reported versus 15% observed hand hygiene in healthcare workers in a large hospital in Vietnam [78]. In the only study that examined it, face covering wearing was self-reported at 25% but observed at 1% in emergency department personnel at a Minnesota public teaching hospital [62].

In only one paper was uptake of protective behaviours lower in self-report data than observed data, and this only applied to a small subset of participants, 4% of the total sample, who expressed that their hand hygiene adherence was between 0 and 10%, compared to an observed rate of 35%. 81% of the total sample rated their hand hygiene adherence as between 80 and 90%, comparable to the 90% adherence that was observed [78].

Self-reported rates of behaviour matched observed rates in three studies, all studying hand hygiene: one study assessing healthcare workers’ in a French university hospital [62], one looking at medical students’ [70], and one looking at healthcare workers’ behaviour in an intensive care unit in South India [80].

### Quality assessment of non COVID-19 studies

**Fig. 3:**
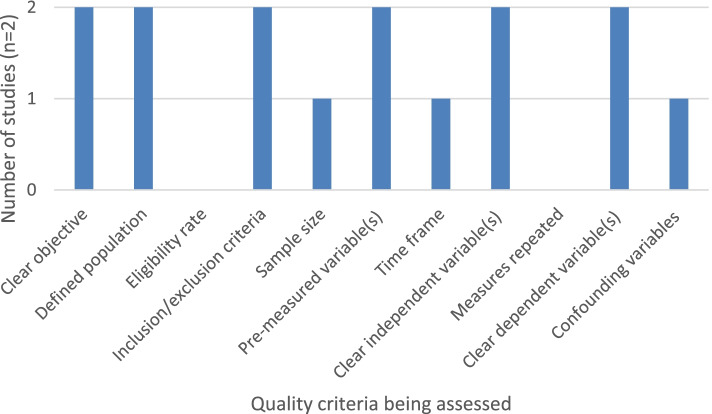
Number of non-COVID-19 intervention studies displaying relevant aspects of NIH quality assessment tool

**Fig. 4:**
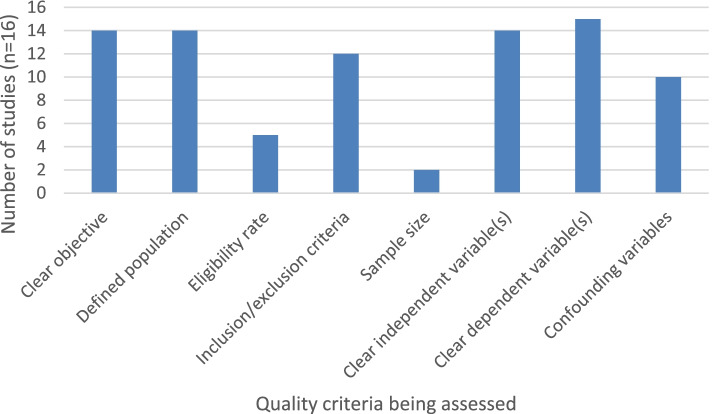
Number of non-COVID-19 non-intervention studies displaying relevant aspects of NIH quality assessment tool

Studies with interventions intended to improve adherence to protective behaviours (*n* = 2) were rated out of 11 relevant criteria on the NIH quality assessment checklist and studies with no interventions (*n* = 16) were rated out of eight relevant criteria (Table S5). Studies with an intervention had a median score of 7, with a range of 6-9 (Fig. 3). Studies without an intervention had a median score of 6, with a range of 1-8 (Fig. 4). Overall, studies in both groups generally had clearly defined study objectives, populations and variables, however very few studies reported any sample size or power estimates.

## Discussion

Improving uptake of health protective behaviours is an important public health challenge, not only for COVID-19, but for infectious disease prevention more widely. Face coverings, hand hygiene and maintaining physical distance have all been identified as effective infection control strategies that have relatively few downsides in comparison to more far-reaching interventions such as society-wide ‘lockdowns’ [11]. Identifying ways to achieve good adherence to such measures requires that we are first able to measure adherence accurately. Though self-report can be a useful proxy for behaviour, our review suggests that academic research has become overly reliant on it, so much so that although we identified 27, 279 papers which included terms related to COVID-19 and to hand hygiene, face covering or social distancing, just 48 of these papers (< 0.2%) actually studied the behaviour in question objectively.

It is likely that several factors influence the way in which behaviour is measured. Speed, cost, ease and the ability to explore associations with other variables which may be best measured via self-report (such as, for example, trust in government, exposure to conspiracy theories or the perceived efficacy of an intervention) are all valid reasons for opting to use self-report over observation. Indeed, the importance of ease as a factor likely explains why only seven studies have attempted to assess physical distancing using observational techniques while 35 have looked at face coverings: it is difficult for an observer to judge distance between two people and to identify whether people need to maintain distance from each other (e.g. if they do or do not live in the same household), but easier to assess whether they are wearing a face covering. Nonetheless, our review also points to the dangers of over-reliance on self-report. The 21 studies that we identified which compared self-report and observed behaviour repeatedly demonstrated that self-report over-estimates hand hygiene behaviour, sometimes dramatically so, while evidence for the validity of self-reported distancing and face covering use is limited in the current literature. Although outside the date range for our search, two recent pre-prints support this point for hand hygiene and extend the evidence of a self-report gap to include face covering and distancing. One study demonstrated that self-reports of “always” wearing a face covering when in specific public spaces in a national UK Government-funded survey matched observed behaviour in those locations, but that an additional 23% of people reported “sometimes” wearing face coverings in these situations, something which could not be accounted for in the observations [80]. The other study, of a single university campus in the UK, found that while 68% of survey respondents reported always cleaning their hands while entering a university building, observation of the only entrance to the main campus building found the true rate to be 16% [81]. Reported and observed rates for distancing were also discordant (49% vs 7%) while the gap for wearing a face covering was smaller but still noticeable (90% vs 82%). While multiple factors may account for these discrepancies [6], recall bias and social desirability would seem most likely to lead to inflated estimates of behaviours such as hand hygiene and physical distancing. Although there are exceptions, with some individuals exercising continuous daily mask use, the relative novelty of face coverings for many people, the limited number of occasions they need to be worn during the day for many members of the general public and their greater salience and hence ease of recall may partly mitigate these biases and explain why self-reported wearing of a covering may be a more reliable measure of behaviour than self-reported hand hygiene or distancing.

Notably, the quality of studies that included an observational measure was generally good in most respects. The one exception was that relatively few studies provided a sample size justification. We suspect this is linked to the difficulty of setting a pre-determined sample size in advance of a naturalistic study. For example, it can be difficult to predict how many people will pass by an observer over a set period of time. The relatively high quality may reflect the tendency for authors who choose to take the difficult route of evaluating behaviour via an objective measure to have also considered other ways of maximising the quality of their study.

### Suggestions for future research

Plenty of scope exists for future work to expand this literature. First, there is a pressing need to establish the validity of self-reported behaviour. At present, the limited literature that exists focusses almost entirely on hand hygiene. During the COVID-19 pandemic we found no studies comparing self-report and observational data for physical distancing and only one for the use of face coverings, although more work in this area is starting to appear [80, 81]. Future work should test approaches to improve the validity of self-report data and also test whether the correlates of self-reported behaviour (which are the basis for many policy recommendations and proposed interventions) can be replicated as correlates of observed behaviour. Consideration should be given to the potential differences in validity that may be observed across population and settings. For example, in the studies that we reviewed, observed adherence tended to be higher in studies of healthcare workers than in general population samples.

Second, our review focussed solely on three behaviours: hand hygiene, face covering use and physical distancing. While important, these are only a subset of the complex set of behaviours that members of the public have been encouraged to adopt during the COVID-19 pandemic. We have not systematically reviewed the literature on the validity of self-report measures of, for example, testing uptake or self-isolation, but have no reason to suspect that self-report is more valid for these behaviours, given that there is substantial social desirability involved and that, for some of them, research participants may technically be liable to legal action if they admit to non-adherence. Nonetheless, key studies on these outcomes rely entirely on self-report [82, 83]. The one notable exception to this list is vaccination, a memorable, binary outcome for which self-report has been shown to be reasonably, though not entirely, valid [5, 84].

Third, while our review may give the impression that observation is a single ‘gold standard’ metric for behaviour, it is clear that there are multiple methods of observation. We identified methods including direct study of behaviours by trained observers, video observation, automated technology, and the use of newly developed technology using AI and machine learning in place of an observer. The use of such technology has been demonstrated with face covering wearing studies, as well as studies that measure crowd density with social distancing within the crowd data [85, 86, 87]. These techniques all have their pros and cons in terms of intrusiveness, cost, capacity, ability to identify behaviours that may be partially obscured and so on. A ‘one-size-fits-all approach’ may not be possible. Nonetheless, further work to develop a set of standardised observational protocols for key outcomes may assist in promoting the use of such techniques and allowing better comparison between studies.

### Limitations

Several limitations should be considered for this systematic review. First, our conclusions are limited by the availability of data in the literature. The relative absence of observational data relating to face covering wearing or physical distancing is an important result in its own right, but also limits our ability to assess the adequacy of self-report for these behaviours. Second, while we made efforts to search widely for relevant studies, including in COVID-19 specific databases, it is possible that we missed some studies which used terminology relating to an observational method that we did not include in our search. Given the rapidity with which the COVID-19 literature has expanded, with approximately a quarter of a million papers appearing in Scopus alone in less than 2 years, it is likely that additional studies will have been added to the databases that we searched in the time taken between completing our search and publication of this paper.

In this review, we have not attempted to pool the rates of behaviour observed in the various studies. The differing contexts in the studies we included means that any pooled estimate would not be meaningful. For example, it is probably not useful to compare rates of observed hand hygiene among healthcare workers working on COVID-19 wards [25] with those among high school students attending their graduations [48].

## Conclusions

The COVID-19 pandemic witnessed an explosion in research covering every aspect of the crisis. Within the field of behavioural science, there has been a heavy focus on ways to promote behaviours believed to reduce infection transmission. Almost all of these studies have measured whether people say they have engaged in specific behaviours. Few have measured the behaviour itself. This is problematic. For hand hygiene, observed adherence tends to be substantially lower than estimates obtained via self-report. There are few studies that have tested the validity of self-reported face covering use or physical distancing, but these also suggest that self-reports tend to be biased. Future research in this field should make greater use of observational methods where possible and should carefully consider the validity of any self-report measure where this is not possible.

## Supplementary Information

**Additional file 1: Figure S1.** Flow chart for included studies aims one and two. **Figure S2.** Flowchart for included studies aim three. **Table S1.** Characteristics of included studies aims one and two (COVID-19 papers). **Table S2.** Characteristics of included studies aim 3 (Non COVID-19). Search Strategy. **Table S3.** COVID-19 Included papers in data synthesis. **Table S4.** NON-COVID-19 papers included in synthesis. **Table S5.** NIH quality assessment checklist.

## Data Availability

Not applicable.
