# Supplementary material for: A systematic review of observational methods used to quantify personal protective behaviours among members of the public during the COVID-19 pandemic, and the concordance between observational and self-report measures in infectious disease health protection
Source: BMC Public Health. 2022 Jul 28;22:1436. doi: 10.1186/s12889-022-13819-0 (PMC9330943; doi:10.1186/s12889-022-13819-0)
Supplement: Supplementary file 1 — Additional file 1: Figure S1. Flow chart for included studies aims one and two. Figure S2. Flowchart for included studies aim three. Table S1. Characteristics of included studies aims one and two (COVID-19 papers). Table S2. Characteristics of included studies aim 3 (Non COVID-19). Search Strategy. Table S3. COVID-19 Included papers in data synthesis. Table S4. NON-COVID-19 papers included in synthesis. Table S5. NIH quality assessment checklist. [file 12889_2022_13819_MOESM1_ESM.docx]

**Supplementary Material**

Figure S1: Flow chart for included studies aims one and two

Figure S2: Flowchart for included studies aim three

Table S1: Characteristics of included studies aims one and two (COVID-19 papers)

|  | **Date of observations** | **Date of publication** | **Intervention component** | **Study design** | **Number of events** | **Sample size** | **Hand hygiene adherence %** | **Face covering adherence %** | **Social distance adherence %** | **Where did observations take place** | **Population observed** | **Other location data** |
| --- | --- | --- | --- | --- | --- | --- | --- | --- | --- | --- | --- | --- |
| 1 | 1 Jan 2015-31 Dec 2020 | Apr-21 | no | direct observer | 13494 | 13494 | 66% | n/a | n/a | hospital | healthcare workers | Sicily, Italy |
| 2 | 22 Apr-9 May 2020 | 13-Aug-20 | no | direct observer | 1000 | 1000 | n/a | 57% | n/a | public parks, banks, outpatient clinics, bus stations | general population | Iran |
| 3 | not reported | 02-Jun-20 | no | direct observer | 382 | 382 | n/a | 64.1% | n/a | hospital | healthcare workers and hospital staff | India |
| 4 | 5-8 Apr 2020 | 09-Jul-20 | no | direct observer | 78 | 78 | n/a | 32% | n/a | food stores | general population | Braga, Portugal |
| 5 | May-Jun 2020 | 18-May-21 | no | video observation | 383 | 383 | n/a | 43% | n/a | public streets | general population | Amsterdam, Netherlands |
| 6 | 5-8 Apr 20; 18-27 Apr 20; 18-27 Jun 2020 | Feb-21 | no | direct observer | 238 | 238 | n/a | 96.6% men,100% women | n/a | food stores | general population | Braga, Portugal |
| 7 | 1-29 Feb 2020 | 25-Mar-21 | no | direct observer | 10211 | 10211 | n/a | 82.5% | n/a | public streets | general population | Hong Kong |
| 8 | 10, 18, 25 May 2020 | 23-Feb-21 | no | direct observer | 3965 | 2353 | n/a | 73.6% at time 1; 66.5% at time 2; 65.7% at time 3; highest while shopping (84.9%; 81.7 and 80.3%) and the lowest during outdoor sport activity (63%; 53.5 and 48.7%). | n/a | public streets, parks, enclosed shopping centres | general population | Poland |
| 9 | Sep-Nov 2020 | 05-Feb-21 | no | direct observer | 17200 | 17200 | n/a | 76.70% | n/a | universities | staff and students | rural and urban universities across USA |
| 10 | 20 Jul 2020 for 4 weeks; 21 Sep 2020 for 14 weeks | 03-May-21 | yes | direct observer | 4122 | 4122 | n/a | 82.2% pre intervention; 92.2% wore after intervention | n/a | hospitals | healthcare workers | Connecticut, USA |
| 11 | Jul-Aug 2020; 23-30 Nov 2020; 1-5 Dec 2020 | 18-Apr-21 | yes | direct observer | 219 | 219 | 35.2%; 40% healthcare workers | 100% of public and healthcare workers | n/a | hospitals | patients and healthcare workers | Bangkok, Thailand |
| 12 | May-20 | 01-Sep-20 | no | direct observer | 271 | 271 | n/a | 94.2% overall. 100% on COVID-19-designated wards and 48% on non-COVID-19 wards | n/a | hospital | healthcare workers | UK |
| 13 | 3-9 Jun 2020; 24 Jul-3 Aug 2020 | 15-Oct-20 | no | direct observer | 9935 | 9935 | n/a | 41.5% | n/a | retail stores | general population | rural and urban Wisconsin, USA |
| 14 | 16-30 May 2020 | 13-Apr-21 | no | direct observer | 1004 | 1004 | n/a | 75.5% | n/a | public businesses | general population | Vermont, USA |
| 15 | 5-8 May 2020 | 03-Aug-20 | no | direct observer | 892 face cover wearing, 871 social distancing | 1641 | n/a | 12.6% | 98% adherence | vehicles | drivers and passengers | Ghana |
| 16 | 30 Mar-12 Apr 2020 | Sep-20 | no | direct observer | 3322 | 3322 | n/a | 98.2% wore face cover; 92.3% wore face cover correctly | n/a | market, hospital | general population | Malaysia |
| 17 | 12 days 9am-8pm during 'phase 2' in Italy | 01-Jan-21 | no | direct observer | 1036 | 1036 | n/a | 25.5% | n/a | in the street', most people observed were outside shops, waiting to enter | general population (excluded children and 'people with special needs' | Italy |
| 18 | 11am to 1pm 6-9 Aug 2020 | 17-Nov-20 | yes | video observation quasi experiment; comparative behavioural observation | 1286 | 400 | n/a | n/a | 20% | a university canteen | university canteen customers | Thailand |
| 19 | 4-25 May 2020 | 03-May-21 | no | direct observer | 182 | 182 | n/a | 94% | n/a | on public transport (subway and local trains) | public transportation users | Paris, France |
| 20 | 21 Sep 2020 to 2 Oct 2020 | 24-Oct-20 | no | direct observer | 187 | 187 | n/a | 80.1% at perimeter, 93.4% by entry | n/a | general ob/gyn outpatient clinic | patients and visitors | North Carolina, USA |
| 21 | 15-18 Jun 2020; 20-21 Jun 2020; 22-24 Jun 2020 | 14-Oct-20 | yes | direct observer- quasi experiment; comparative behavioural observation | 466 | 466 | 0% adherence pre intervention; 12% adherence post intervention | n/a | n/a | hospital | outpatients and visitors | Thailand |
| 22 | 2-11 Aug 2020 | 14-Jan-21 | no | direct observer | 10440 | 10440 | n/a | 45.6% wore face cover; 75.6% of face covering wearers wore it correctly | n/a | in the street | general population | 8 urban districts, 92 neighbourhoods of Ahvaz, Iran |
| 23 | Jan 2018-October 2019; Feb-Mar 2020 | 29-Jul-20 | no | video observation | 7586 | 7586 | n/a | face cover wearing increased in all regions except the US, from (1.1%) to (99.4%) in mainland China.(3.1%) to (38.7%) in Japan (0.8%) to (85.5% ) in South Korea. (0.2%) to (1.6%) in Western Europe and (0.4%) to (2.1%) in the US | n/a | public transportation stations, streets, parks | general population | China, Japan, South Korea, US, England, France, Germany, Spain, Italy |
| 24 | 5-7 Mar 2020 | 06-Jun-20 | no | direct observer | 1761 | n/a | 79.44% | n/a | n/a | hospital | healthcare workers | Tongji, China |
| 25 | 27 Feb-21 Apr 2020 | 10-Dec-20 | no | direct observer | 610 | 127 | 82% adherence in COVID-19ward vs 65% non-COVID-19 ward | n/a | n/a | hospital pulmonology department | healthcare workers | Cologne, Germany |
| 26 | 21-30 Apr 2020 | 26-Oct-20 | no | direct observer | 468 | 468 | n/a | 85.42% wore face covering; 66% wore face covering correctly; | n/a | street, public transportation, grocery store line | general population | Basseterre, St. Kitts |
| 27 | Jul-Aug 2020 | 12-Mar-21 | no | direct observer | 422 | 422 | n/a | 54.68% | n/a | taxi stands | taxi drivers | Dessie City and Kombolcha Towns, Ethiopia |
| 28 | 20-24 Jul 2020 | 28-Dec-20 | no | direct observer | 2302 | 2286 | n/a | 98% | n/a | ophthalmology outpatient clinics | patients | Pennsylvania, USA |
| 29 | Apr-Jun 2020 | 03-May-21 | no | direct observer | 2080 | 2080 | n/a | 65% | n/a | outside grocery stores | general population | Western Australia |
| 30 | Jul-20 | 05-Jan-21 | no | direct observer | 1096 | 1096 | n/a | 70.2% at mosque; 68.7% in malls; 32.6% in park; 45.2% in barbershop | n/a | in the community (mosques, malls, parks, barbershops) | general population | cities in Jazan region of Saudi Arabia |
| 31 | 30-Jul-20 | 01-Sep-20 | no | direct observer | 200 | 100 | n/a | 77% | n/a | outdoor street locations | general population | Waikiki and Honolulu, Hawaii, USA |
| 32 | Jun-20 | 18-Nov-20 | no | direct observer | 431 | 431 | 57.46% adherence on subway 44.78% adherence when shopping | 78.35% on subway; 46.01% when shopping | n/a | subway station and a shop | general population | Tehran, Iran |
| 33 | 25 Jun-21 Jul 2020 | 29-Mar-21 | no | direct observer | 3354 | 3354 | n/a | 56.4% wore face coverings; 75.2% of face coverings were worn correctly | n/a | inside shopping centres and train stations; outside shopping centres | general population | 13 cities in Pays de la Loire, France |
| 34 | 4 May-11 Jun 2020 | 25-Jan-21 | no | direct observer | 3061 | 3061 | 2% | 96% smokers possessed a face covering. While smoking, 0.2% wore there face covering fully, 81.6% of smokers put the face cover under the chin and 13.8% carried it in the hand, 32.4% did not wear a face covering immediately after smoking. | 25.7% | outdoor street locations | general population | Hong Kong |
| 35 | Jul-20 | 24-Jan-21 | no | mixed methods. direct observer/media | 1152 | 1152 people | n/a | 70% | 0 | in person high school graduation | high school students | USA |
| 36 | unstated, during COVID-19 | Dec-20 | no | video observation | 1594 | 1594 | n/a | n/a | 40.4% Oxford Town Centre dataset; 17.6% in the Mall dataset; and, 58.1% CUHK Square dataset | oxford town centre | all general public any age | city centre/shopping mall UK |
| 37 | 2 January to 15 March 2020 | Oct-21 | no | direct observer | 267 | unknown | 47 per cent pre-COVID-19 pandemic to 95 per cent just before lockdown | n/a | n/a | obstetric ward | healthcare worker | Germany |
| 38 | unknown | Dec-20 | no | direct observer and survey | 400 | 400 people | 45.6% | n/a | n/a | home health care | nurses | USA |
| 39 | June 1, 2020, to July 1, 2020 | Oct-20 | yes | direct observer | 42 | 42 people | 33.59% | n/a | n/a | tertiary care hospital | surgical trainees | Calcutta |
| 40 | unknown | Jan-21 | no | direct observer | 1053 | unknown | 15% | 68.2% | n/a | private and government-subsidized RCHs | healthcare worker | Hong Kong |
| 41 | February 7 to 25, 2020 | 2020 | no | direct observer | 11680 | 12208 people | n/a | for women, 70.5% and men 50% | n/a | residential areas, shopping districts, office quarters | adults and children over 2 in general population. | Taipei and major satellite cities in New Taipei |
| 42 | 10 weeks before the time of the school closures (the week beginning January 5 through the week beginning March 8) with the 10-week period after school closures (the week beginning March 15 through the week beginning May 17). | Jan-21 | no | automated technology hand sanitizer event measurement | 35,362,136 | unknown | 48.52% before the school closures to 58.05% after | n/a | n/a | 9 US hospitals | healthcare workers/general public | USA |
| 43 | Mar 2020 - Jun 2020) | Nov-20 | no | mixed methods. automated technology hand sanitizer event measurement and observer | 593,118 | unknown | 85% hospital wide and 90% on COVID-19 wards | n/a | n/a | inpatient areas of the hospital | healthcare workers | USA |
| 44 | April to July 2020 | Apr-21 | yes | direct observer | 60 | 60 | n/a | n/a | 78.3% | regional hospital in Korea. | healthcare workers | Korea |
| 45 | March and April 2020 | April 2020 | no | direct observer | unknown | unknown | 97% | n/a | n/a | tertiary care hospital Pakistan | healthcare workers | Pakistan |
| 46 | Oct-20 | Jul-20 | no | direct observer | 300 | 300 | 85% | 74% | 86% | paediatric tertiary care centre | parents of children on arrival to hospital | India |
| 47 | unknown | Sep-20 | no | direct observer | 400 | 48 | 45.6%. highest after contact with body fluid (65.1%) and lowest after touching a patient (29.5%). | n/a | n/a | home health care | healthcare workers | USA |
| 48 | Jul-20 | Feb-21 | no | video recording | 780 | 780 | n/a | 61% | 69% | Arches National Park | general public | USA |

**Table S2: Characteristics of included studies aim 3 (Non COVID-19)**

| **Study** | **Date of observation** | **Date of pub** | **Study design** | **Number of events** | **Sample size** | **Hand hygiene adherence observed** | **Self-reported**  **prevalence** | **Where did observations take place** | **Population observed** | **Other location data** |
| --- | --- | --- | --- | --- | --- | --- | --- | --- | --- | --- |
| 1 | unknown | 2006 | direct observer/survey | 890 | 60 | period 1, student hand hygiene was 49.1%, mentors 44.3 % during observational period 2, student hand hygiene was 52.3%, mentor 53.3%. | students reported 92.98 before patient contact, 95.53 for commitment to handwashing after patient contact, 84.89 before donning gloves, and 95.11 after removal of gloves.  Based on a scale of 10-100. 10 being lowest possible. | hospital setting | sixty students enrolled in a certified nursing program were selected to participate in the study. | USA |
| 2 | unknown but questionnaire was 1 year before observations | 2002 | questionnaire, observation and hand bacteria measurement | 555 observed hand hygiene opportunities | 13 observed, 51 survey responses (from a conference - not the same people) | 31.4% of all opportunities; 53.9% 1-5 times a day; 38.5% only after contact with infected material; 7.7% before each physical examination | 37.3% following contact with contaminant; 35.3% prior to every patient examination; 27.5% 1-5 times a day 3.9% following every patient exam; 3.9% once per hour | two outpatient dermatology clinics | physicians | Israel |
| 3 | October 1999-April 2000 | 2001 | questionnaire, observation | not reported | 61 artists surveyed, 25 of whom were observed | 71% | 83% | 29 professional tattoo studios | professional tattooists | the seven-county metropolitan area of Minneapolis and St. Paul, Minnesota |
| 4 | Sep-02 | Nov-04 | questionnaire, observation | not reported | 1050 survey responses, 206 observed | 74% | 74% | 25 care units within a University Hospital | medical staff (physicians, nurses, nursing assistants) | Nantes University Hospital, France |
| 5 | July-Sept 1989 | Jan-92 | questionnaire, observation | 1,018 procedures were reported x 4 outcomes = 4072 | 66 survey responses, 88 staff working (not reported how many were observed) | face covering 1.2%; | face coverings: 25.5%; | emergency department of a university hospital | medical staff (12 staff physicians, five to ten resident physicians, physician assistants registered nurses, nursing assistants and other ancillary and support staff) healthcare workers | urban, the Minneapolis/StPaul (twin cities) metropolitan area |
| 6 | 2016 observations, 6 months later (2017) interviews | 2019 | interview and observation | 4957 hand hygiene opportunities | 25 interviews, observed not stated | hand hygiene before gloving was performed 42% | 88% | 3 teaching hospitals, including 33 units (haemodialysis units, paediatric wards, intensive care units (ICUs), and emergency departments (EDs) | (physician, physician assistant, nurse, nursing assistant, nurse practitioner) | eastern and midwestern USA |
| 7 | Nov-13 | March-May 2012 | mixed methods, self-report and direct observed by observer | 91 | 51 | hand hygiene before glove application, was present in 14% of the CMCs and 21% of the MDAMCs | hand hygiene before glove application CMCs (63%) MDAMCs (50%). hand hygiene on room exit CMCs (84%) MDAMCs (94%) | 3 medical/surgical units at 2 medical centres within the south-eastern United States. One was a 44-bed medical/surgical unit at a 110-bed community medical centre (CMC) and the other 2 units were 28-bed medical/surgical units at a 600 bed magnet-designated academic medical centre (MDAMC) | registered nurses comprised 78% of the total sample of respondents. Although physicians and therapy providers were invited to participate, none completed the survey. Therefore, results focus on licensed and unlicensed nursing staff. | USA |
| 8 | unknown | Feb-19 | mixed methods: direct observer and survey | 997 | 371 survey participants. 997 people observed | physicians had the lowest rate of compliance at 14.6%, while nurses had the highest rate of compliance at 38.8%. | physicians had the lowest rate (67.2%) of self-reported hand hygiene compliance, while nurses had the highest rate (97.8%), | 11 inpatient service departments were chosen for the study: Five internal medicine services, five paediatrics services, and one intensive care unit service | the subjects of the study were healthcare workers of different positions: physicians, nurses, care assistants, and student nurses. | Vietnam |
| 9 | April and June 2016 | May 2019 | direct observers and survey | 811 opportunities observed. A total of 87 questionnaires were returned | 87 | overall hand hygiene compliance was 56% during baseline and 64% during intervention period, respectively. | self-reported hand hygiene compliance did not differ between baseline and intervention (4.12 vs 4.03) on a Likert scale 1-5, 5 being always | University Hospital Zurich | healthcare workers | Switzerland |
| 10 | July-August 2016 | May-19 | mixed methods: direct observer and survey, interviews | 302. | 104 observed, 218 survey | hand hygiene was 35% of all opportunities. Overall entry and exit hand compliance was 90% | 81% of the sample estimating they miss performing hand hygiene when they realize it should be performed 10%-20% of the time. An additional 11% reported missing hand hygiene 30%-40% of the time. 4% estimated they missed performing hand hygiene 90%-100% of the time. | ICU at the University of Maryland Medical Center, a 750-bed tertiary care hospital in Baltimore, Maryland. | healthcare workers | USA |
| 11 | unknown | Oct-18 | mixed methods, self-report and direct observed by observer | 249 observed hand hygiene | 87 participants (46 medical doctors, 21 from the public hospital, 9 from the private clinic, and 16 from the Security Forces; and 41 nurses, 14 from the public hospital, 9 from the private clinic, and 18 from the security forces hospital) were recruited. Of the 87 participants who completed the questionnaire, 83 participants were observed during exams with at least three patients and hand hygiene practices were recorded using the checklist | physicians (27%) and nurses (29%) | Likert scale. Physicians mean self-report was 10.19/15 based on 3 hand hygiene opportunities, and nurses was 13.65/15 based on 3 opportunities. | outpatient examination rooms and emergency departments of three types of hospitals | 87 physicians and nurses recruited while on duty during the scheduled observation periods, with each healthcare worker being observed during individual medical examinations with at least three patients | Eastern region of Saudi Arabia |
| 12 | unknown | 2011 | survey self-report vs direct observation | unknown | 160 medical students in year 3 and 98 in year 4 | direct observations reported proper hand hygiene in 87.9% | 87.90% | hospital setting | medical students | USA |
| 13 | fourth quarter of 2011 to 82.3% in fourth quarter of 2012 | Jun-13 | mixed methods, self-report and direct observed by observer | unknown | unknown | the overall hand hygiene compliance rate increased from a baseline of 49.7% in fourth quarter of 2011 to 82.3% in fourth quarter of 2012 | increased from a baseline of 82.9% to 93.8% | healthcare settings | healthcare workers doctors | Korea |
| 14 | Aug-12 | 2013 | mixed methods, self-report and direct observed by observer | 470 minutes of observations in total | the observed population was 13% doctors, 70% nurses, 9% housekeeping staff, 9% visitors of patients. Questionnaires were 35 nurses and 34 doctors. | nurses: 47% actual observed compliance with hand hygiene, doctors: 51% actual observed compliance | nurses: 88% compliance with hand hygiene, doctors: 85% compliance | modern oncology hospital | medical staff (nurses, doctors) and some housekeeping staff and visitors | India |
| 15 | unknown | 2016 | direct observer/survey | 457 | 102 | 29% overall compliance. post-test compliance increased to 51 % | “i wash my hands after using the rest room” was rated a median of 5/5 on a Likert scale, quartiles 4-5 before and after intervention | hospital setting | (healthcare workers, comprising 76 radiographers,17 nurses, and nine healthcare assistants (HCA), agreed to participate in the study. | Hong Kong |
| 16 | the baseline survey was held from May through August 2008 and covered a total of 3576 households. The follow-up survey was conducted following the completion of the project activities (March–June 2011). | Oct-15 | survey self-report vs direct observation | unknown | 3576 in survey, 45-60 households observed | handwashing with soap was observed in only 16% of the events that required it. 20% of faecal contact events, 25% of eating events, 6% of child feeding events, and 10% of food preparation events. | although almost all caregivers reported having washed their hands with soap at least once during the previous 24 h, fewer than half conﬁrmed having carried out so at times of faecal contact (39% of caregivers associated handwashing with soap with toilet use and 34% with cleaning up after children). cooking or food preparation (68%), but lower when feeding a child (31%). | households | general public | Peru |
| 17 | unknown | Jun-15 | survey self-report vs direct observation | unknown | sixty nine respondents participated in the survey which predominantly included the nurses | use of alcohol based hand rub 94.2%, five moments of hand hygiene 88.5%, 6 steps of hand hygiene 65%. | use of alcohol based hand rub 98.5, five moments of hand hygiene Q 88.5, 6 steps of hand hygiene Q-92.5 | in ICU in an oncology, BMT and neurosurgical centre in South India | healthcare workers mostly nurses | South India |
| 18 | 2015-2016 | Feb-2019 | survey self-report vs direct observation | 127 | 639 ,127 of these healthcare workers had been also directly observed. | 67.7% | 74% | hospital setting | healthcare workers physicians and nurses | 5 hospitals in Slovakia and 3 in Czech republic |

**Search Strategy**

EMBASE SEARCH COVID-19 papers Aim 1 and 2

1. exp coronavirus disease 2019/

2. exp Severe acute respiratory syndrome coronavirus 2/

3. exp Coronavirinae/

4. COVID-19-19.mp. [mp=title, abstract, heading word, drug trade name, original title, device manufacturer, drug manufacturer, device trade name, keyword, floating subheading word, candidate term word]

5. coronavirus.mp. [mp=title, abstract, heading word, drug trade name, original title, device manufacturer, drug manufacturer, device trade name, keyword, floating subheading word, candidate term word]

6. COVID-19.mp. [mp=title, abstract, heading word, drug trade name, original title, device manufacturer, drug manufacturer, device trade name, keyword, floating subheading word, candidate term word]

7. 1 or 2 or 3 or 4 or 5 or 6

8. exp hand washing/

9. (hand adj3 wash*).mp. [mp=title, abstract, heading word, drug trade name, original title, device manufacturer, drug manufacturer, device trade name, keyword, floating subheading word, candidate term word]

10. (hand adj3 hygiene).mp. [mp=title, abstract, heading word, drug trade name, original title, device manufacturer, drug manufacturer, device trade name, keyword, floating subheading word, candidate term word]

11. (hand adj3 sanit*).mp. [mp=title, abstract, heading word, drug trade name, original title, device manufacturer, drug manufacturer, device trade name, keyword, floating subheading word, candidate term word]

12. (hand adj3 disinect*).mp. [mp=title, abstract, heading word, drug trade name, original title, device manufacturer, drug manufacturer, device trade name, keyword, floating subheading word, candidate term word]

13. hand-sanit*.mp. [mp=title, abstract, heading word, drug trade name, original title, device manufacturer, drug manufacturer, device trade name, keyword, floating subheading word, candidate term word]

14. (hand adj3 clean*).mp. [mp=title, abstract, heading word, drug trade name, original title, device manufacturer, drug manufacturer, device trade name, keyword, floating subheading word, candidate term word]

15. hand-wash*.mp. [mp=title, abstract, heading word, drug trade name, original title, device manufacturer, drug manufacturer, device trade name, keyword, floating subheading word, candidate term word]

16. hand-hygiene.mp. [mp=title, abstract, heading word, drug trade name, original title, device manufacturer, drug manufacturer, device trade name, keyword, floating subheading word, candidate term word]

17. 8 or 9 or 10 or 11 or 12 or 13 or 14 or 15 or 16

18. exp face mask ventilation/ or exp mask/ or exp cloth mask/ or exp face mask/ or exp pediatric face mask/ or exp surgical mask/

19. (face adj3 cover*).mp. [mp=title, abstract, heading word, drug trade name, original title, device manufacturer, drug manufacturer, device trade name, keyword, floating subheading word, candidate term word]

20. mask*.mp.

21. (wear adj3 mask).mp. [mp=title, abstract, heading word, drug trade name, original title, device manufacturer, drug manufacturer, device trade name, keyword, floating subheading word, candidate term word]

22. (facial adj3 cover*).mp. [mp=title, abstract, heading word, drug trade name, original title, device manufacturer, drug manufacturer, device trade name, keyword, floating subheading word, candidate term word]

23. (facial adj3 mask).mp. [mp=title, abstract, heading word, drug trade name, original title, device manufacturer, drug manufacturer, device trade name, keyword, floating subheading word, candidate term word]

24. facial-cover*.mp. [mp=title, abstract, heading word, drug trade name, original title, device manufacturer, drug manufacturer, device trade name, keyword, floating subheading word, candidate term word]

25. (wore adj3 mask).mp. [mp=title, abstract, heading word, drug trade name, original title, device manufacturer, drug manufacturer, device trade name, keyword, floating subheading word, candidate term word]

26. 18 or 19 or 20 or 21 or 22 or 23 or 24 or 25

27. exp social distancing/

28. (social* adj3 distanc*).mp. [mp=title, abstract, heading word, drug trade name, original title, device manufacturer, drug manufacturer, device trade name, keyword, floating subheading word, candidate term word]

29. (physical* adj3 distanc*).mp. [mp=title, abstract, heading word, drug trade name, original title, device manufacturer, drug manufacturer, device trade name, keyword, floating subheading word, candidate term word]

30. (safe adj3 distanc*).mp. [mp=title, abstract, heading word, drug trade name, original title, device manufacturer, drug manufacturer, device trade name, keyword, floating subheading word, candidate term word]

31. 27 or 28 or 29 or 30

32. CCTV.mp.

33. (closed adj3 circuit adj3 television).mp. [mp=title, abstract, heading word, drug trade name, original title, device manufacturer, drug manufacturer, device trade name, keyword, floating subheading word, candidate term word]

34. exp videorecording/

35. (surveillance adj3 camera).mp. [mp=title, abstract, heading word, drug trade name, original title, device manufacturer, drug manufacturer, device trade name, keyword, floating subheading word, candidate term word]

36. 32 or 33 or 34 or 35

37. exp observational study/ or exp observational method/

38. observ*.mp. [mp=title, abstract, heading word, drug trade name, original title, device manufacturer, drug manufacturer, device trade name, keyword, floating subheading word, candidate term word]

39. 37 or 38

40. 17 or 26 or 31

41. 7 and 40

42. 39 and 41

43. 7 and 36 and 40

44. 42 or 43

MEDLINE SEARCH COVID-19 papers Aim 1 and 2

1. exp COVID-19-19/

2. exp SARS-CoV-2/

3. exp Coronavirus/

4. COVID-19-19.mp. [mp=title, abstract, original title, name of substance word, subject heading word, floating sub-heading word, keyword heading word, organism supplementary concept word, protocol supplementary concept word, rare disease supplementary concept word, unique identifier, synonyms]

5. coronavirus.mp. [mp=title, abstract, original title, name of substance word, subject heading word, floating sub-heading word, keyword heading word, organism supplementary concept word, protocol supplementary concept word, rare disease supplementary concept word, unique identifier, synonyms]

6. COVID-19.mp. [mp=title, abstract, original title, name of substance word, subject heading word, floating sub-heading word, keyword heading word, organism supplementary concept word, protocol supplementary concept word, rare disease supplementary concept word, unique identifier, synonyms]

7. 1 or 2 or 3 or 4 or 5 or 6

8. exp Hand Disinfection/

9. (hand adj3 wash*).mp. [mp=title, abstract, original title, name of substance word, subject heading word, floating sub-heading word, keyword heading word, organism supplementary concept word, protocol supplementary concept word, rare disease supplementary concept word, unique identifier, synonyms]

10. (hand adj3 hygiene).mp. [mp=title, abstract, original title, name of substance word, subject heading word, floating sub-heading word, keyword heading word, organism supplementary concept word, protocol supplementary concept word, rare disease supplementary concept word, unique identifier, synonyms]

11. (hand adj3 sanit*).mp. [mp=title, abstract, original title, name of substance word, subject heading word, floating sub-heading word, keyword heading word, organism supplementary concept word, protocol supplementary concept word, rare disease supplementary concept word, unique identifier, synonyms]

12. (hand adj3 disinfect*).mp. [mp=title, abstract, original title, name of substance word, subject heading word, floating sub-heading word, keyword heading word, organism supplementary concept word, protocol supplementary concept word, rare disease supplementary concept word, unique identifier, synonyms]

13. hand-sanit*.mp. [mp=title, abstract, original title, name of substance word, subject heading word, floating sub-heading word, keyword heading word, organism supplementary concept word, protocol supplementary concept word, rare disease supplementary concept word, unique identifier, synonyms]

14. (hand adj3 clean*).mp. [mp=title, abstract, original title, name of substance word, subject heading word, floating sub-heading word, keyword heading word, organism supplementary concept word, protocol supplementary concept word, rare disease supplementary concept word, unique identifier, synonyms]

15. hand-wash*.mp. [mp=title, abstract, original title, name of substance word, subject heading word, floating sub-heading word, keyword heading word, organism supplementary concept word, protocol supplementary concept word, rare disease supplementary concept word, unique identifier, synonyms]

16. hand-hygiene.mp. [mp=title, abstract, original title, name of substance word, subject heading word, floating sub-heading word, keyword heading word, organism supplementary concept word, protocol supplementary concept word, rare disease supplementary concept word, unique identifier, synonyms]

17. 8 or 9 or 10 or 11 or 12 or 13 or 14 or 15 or 16

18. exp Masks/

19. (face adj3 cover).mp. [mp=title, abstract, original title, name of substance word, subject heading word, floating sub-heading word, keyword heading word, organism supplementary concept word, protocol supplementary concept word, rare disease supplementary concept word, unique identifier, synonyms]

20. mask*.mp. [mp=title, abstract, original title, name of substance word, subject heading word, floating sub-heading word, keyword heading word, organism supplementary concept word, protocol supplementary concept word, rare disease supplementary concept word, unique identifier, synonyms]

21. (wear adj3 mask).mp. [mp=title, abstract, original title, name of substance word, subject heading word, floating sub-heading word, keyword heading word, organism supplementary concept word, protocol supplementary concept word, rare disease supplementary concept word, unique identifier, synonyms]

22. (facial adj3 cover*).mp. [mp=title, abstract, original title, name of substance word, subject heading word, floating sub-heading word, keyword heading word, organism supplementary concept word, protocol supplementary concept word, rare disease supplementary concept word, unique identifier, synonyms]

23. (facial adj3 mask).mp. [mp=title, abstract, original title, name of substance word, subject heading word, floating sub-heading word, keyword heading word, organism supplementary concept word, protocol supplementary concept word, rare disease supplementary concept word, unique identifier, synonyms]

24. facial-cover*.mp. [mp=title, abstract, original title, name of substance word, subject heading word, floating sub-heading word, keyword heading word, organism supplementary concept word, protocol supplementary concept word, rare disease supplementary concept word, unique identifier, synonyms]

25. (wore adj3 mask).mp. [mp=title, abstract, original title, name of substance word, subject heading word, floating sub-heading word, keyword heading word, organism supplementary concept word, protocol supplementary concept word, rare disease supplementary concept word, unique identifier, synonyms]

26. 18 or 19 or 20 or 21 or 22 or 23 or 24 or 25

27. exp Physical Distancing/

28. (social* adj3 distanc*).mp. [mp=title, abstract, original title, name of substance word, subject heading word, floating sub-heading word, keyword heading word, organism supplementary concept word, protocol supplementary concept word, rare disease supplementary concept word, unique identifier, synonyms]

29. (physical* adj3 distanc*).mp. [mp=title, abstract, original title, name of substance word, subject heading word, floating sub-heading word, keyword heading word, organism supplementary concept word, protocol supplementary concept word, rare disease supplementary concept word, unique identifier, synonyms]

30. (safe adj3 distanc*).mp. [mp=title, abstract, original title, name of substance word, subject heading word, floating sub-heading word, keyword heading word, organism supplementary concept word, protocol supplementary concept word, rare disease supplementary concept word, unique identifier, synonyms]

31. 27 or 28 or 29 or 30

32. exp Video Recording/

33. CCTV.mp. [mp=title, abstract, original title, name of substance word, subject heading word, floating sub-heading word, keyword heading word, organism supplementary concept word, protocol supplementary concept word, rare disease supplementary concept word, unique identifier, synonyms]

34. (closed adj3 circuit adj3 television).mp. [mp=title, abstract, original title, name of substance word, subject heading word, floating sub-heading word, keyword heading word, organism supplementary concept word, protocol supplementary concept word, rare disease supplementary concept word, unique identifier, synonyms]

35. (surveillance adj3 camera).mp. [mp=title, abstract, original title, name of substance word, subject heading word, floating sub-heading word, keyword heading word, organism supplementary concept word, protocol supplementary concept word, rare disease supplementary concept word, unique identifier, synonyms]

36. 32 or 33 or 34 or 35

37. exp Observational Study/

38. observ*.mp. [mp=title, abstract, original title, name of substance word, subject heading word, floating sub-heading word, keyword heading word, organism supplementary concept word, protocol supplementary concept word, rare disease supplementary concept word, unique identifier, synonyms]

39. 37 or 38

40. 17 or 26 or 31

41. 7 and 40

42. 39 and 41

43. 7 and 36 and 40

44. 42 or 43

SEARCH STRATEDGY Aim 3

1. exp hand washing/

2. (hand adj3 wash*).mp. [mp=title, abstract, heading word, drug trade name, original title, device manufacturer, drug manufacturer, device trade name, keyword, floating subheading word, candidate term word]

3. (hand adj3 hygiene).mp. [mp=title, abstract, heading word, drug trade name, original title, device manufacturer, drug manufacturer, device trade name, keyword, floating subheading word, candidate term word]

4. (hand adj3 sanit*).mp. [mp=title, abstract, heading word, drug trade name, original title, device manufacturer, drug manufacturer, device trade name, keyword, floating subheading word, candidate term word]

5. (hand adj3 disinect*).mp. [mp=title, abstract, heading word, drug trade name, original title, device manufacturer, drug manufacturer, device trade name, keyword, floating subheading word, candidate term word]

6. hand-sanit*.mp. [mp=title, abstract, heading word, drug trade name, original title, device manufacturer, drug manufacturer, device trade name, keyword, floating subheading word, candidate term word]

7. (hand adj3 clean*).mp. [mp=title, abstract, heading word, drug trade name, original title, device manufacturer, drug manufacturer, device trade name, keyword, floating subheading word, candidate term word]

8. hand-wash*.mp. [mp=title, abstract, heading word, drug trade name, original title, device manufacturer, drug manufacturer, device trade name, keyword, floating subheading word, candidate term word]

9. hand-hygiene.mp. [mp=title, abstract, heading word, drug trade name, original title, device manufacturer, drug manufacturer, device trade name, keyword, floating subheading word, candidate term word]

10. 1 or 2 or 3 or 4 or 5 or 6 or 7 or 8 or 9

11. exp face mask ventilation/ or exp mask/ or exp cloth mask/ or exp face mask/ or exp pediatric face mask/ or exp surgical mask/

12. (face adj3 cover*).mp. [mp=title, abstract, heading word, drug trade name, original title, device manufacturer, drug manufacturer, device trade name, keyword, floating subheading word, candidate term word]

13. mask*.mp.

14. (wear adj3 mask).mp. [mp=title, abstract, heading word, drug trade name, original title, device manufacturer, drug manufacturer, device trade name, keyword, floating subheading word, candidate term word]

15. (facial adj3 cover*).mp. [mp=title, abstract, heading word, drug trade name, original title, device manufacturer, drug manufacturer, device trade name, keyword, floating subheading word, candidate term word]

16. (facial adj3 mask).mp. [mp=title, abstract, heading word, drug trade name, original title, device manufacturer, drug manufacturer, device trade name, keyword, floating subheading word, candidate term word]

17. facial-cover*.mp. [mp=title, abstract, heading word, drug trade name, original title, device manufacturer, drug manufacturer, device trade name, keyword, floating subheading word, candidate term word]

18. (wore adj3 mask).mp. [mp=title, abstract, heading word, drug trade name, original title, device manufacturer, drug manufacturer, device trade name, keyword, floating subheading word, candidate term word]

19. 11 or 12 or 13 or 14 or 15 or 16 or 17 or 18

20. exp social distancing/

21. (social* adj3 distanc*).mp. [mp=title, abstract, heading word, drug trade name, original title, device manufacturer, drug manufacturer, device trade name, keyword, floating subheading word, candidate term word]

22. (physical* adj3 distanc*).mp. [mp=title, abstract, heading word, drug trade name, original title, device manufacturer, drug manufacturer, device trade name, keyword, floating subheading word, candidate term word]

23. (safe adj3 distanc*).mp. [mp=title, abstract, heading word, drug trade name, original title, device manufacturer, drug manufacturer, device trade name, keyword, floating subheading word, candidate term word]

24. 20 or 21 or 22 or 23

25. CCTV.mp.

26. (closed adj3 circuit adj3 television).mp. [mp=title, abstract, heading word, drug trade name, original title, device manufacturer, drug manufacturer, device trade name, keyword, floating subheading word, candidate term word]

27. exp videorecording/

28. (surveillance adj3 camera).mp. [mp=title, abstract, heading word, drug trade name, original title, device manufacturer, drug manufacturer, device trade name, keyword, floating subheading word, candidate term word]

29. 25 or 26 or 27 or 28

30. exp observational study/ or exp observational method/

31. observ*.mp. [mp=title, abstract, heading word, drug trade name, original title, device manufacturer, drug manufacturer, device trade name, keyword, floating subheading word, candidate term word]

32. exp infection/

33. 10 or 19 or 24

34. 32 and 33

35. 30 or 31

36. 29 or 35

37. 34 and 36

**Table S3: COVID-19 Included papers in data synthesis**

|  | Title | Journal | Authors |
| --- | --- | --- | --- |
| 1 | Has the COVID-19Virus Changed Adherence to Hand Washing among Healthcare workers?. | Behav Sci (Basel) | Ragusa, Rosalia; Marranzano, Marina; Lombardo, Alessandro; Quattrocchi, Rosalba; Bellia, Maria Alessandra; Lupo, Lorenzo |
| 2 | Face touching in the time of COVID-19in Shiraz, Iran | Am. J. Infect. Control | Shiraly, Ramin; Shayan, Zahra; McLaws, Mary-Louise |
| 3 | Rational use of face mask in a tertiary care hospital setting during COVID-19pandemic: An observational study | Indian J Public Health | Supehia, Sakshi; Sharma, Twinkle; Singh, Vanya; Khapre, Meenakshi; Gupta, Puneet Kumar |
| 4 | Prevention measures for COVID-19in retail food stores in Braga, Portugal | Pulmonology | Precioso, J.; Samorinha, C.; Alves, R. |
| 5 | Face-touching behaviour as a possible correlate of mask-wearing: A video observational study of public place incidents during the COVID-19pandemic | Transboundary Emer. Dis. | Liebst, Lasse S.; Ejbye-Ernst, Peter; Thomas, Josephine; de Bruin, Marijn; Lindegaard, Marie R. |
| 6 | Prevention of COVID-19in retail food stores in Portugal: The importance of regulations in behavioural change | Aten. Prim. | Precioso, Jose; Samorinha, Catarina |
| 7 | Behavioural insights and attitudes on community masking during the initial spread of COVID-19in Hong Kong | Hong Kong Med. J. | Tam, Victor C. W.; Tam, S.Y.; Law, Helen K. W.; Lee, Shara W. Y.; Khaw, M.L.; Chan, Catherine P. L. |
| 8 | Use of masks in public places in Poland during SARS-Cov-2 epidemic: a covert observational study | BMC Public Health | Ganczak, Maria; Pasek, Oskar; Duda-Duma, Lukasz; Swistara, Dawid; Korzen, Marcin |
| 9 | Observed Face Mask Use at Six Universities - United States, September-November 2020 | MMWR Morb Mortal Wkly Rep | Barrios, Lisa C.; Riggs, Margaret A.; Green, Ridgely Fisk; Czarnik, Michaila; Nett, Randall J.; Staples, J Erin; Welton, Michael David; Muilenburg, Jessica Legge; Zullig, Keith J.; Gibson-Young, Linda; Perkins, Andrea V.; Prins, Cindy; Lauzardo, Michael; Shapiro, Jerne; Asimellis, George; Kilgore-Bowling, Genesia; Ortiz-Jurado, Kenny; Gutilla, Margaret J. |
| 10 | Increasing Facemask Compliance among Healthcare Personnel during the COVID-19Pandemic | Infect. Control Hosp. Epidemiol. | Pellegrino, Anthony; Datta, Rupak; Glenn, Keith; Tuan, Jessica; Kayani, Jehanzeb; Patel, Kavin; Fisher, Ann; Linde, Brian; Calo, Lisbeysi; Dembry, Louise Marie |
| 11 | Improving knowledge, attitudes and practice to prevent COVID-19transmission in Healthcare workers and the public in Thailand | BMC Public Health | Skuntaniyom, Sumawadee; Muntajit, Thanomvong; Malathum, Kumtorn; Maude, Rapeephan R.; Jongdeepaisal, Monnaphat; Khuenpetch, Worarat; Taleangkaphan, Keetakarn; Blacksell, Stuart D.; Pan-Ngum, Wirichada; Maude, Richard James |
| 12 | An observational study to identify types of personal protective equipment breaches on inpatient wards | J. Hosp. Infect. | Avo, C.; Cawthorne, K.-R.; Walters, J.; Healy, B. |
| 13 | Who is wearing a mask? Gender-, age-, and location-related differences during the COVID-19pandemic | PLoS ONE | Hart, Meggie Rose; Opielinski, Lauren; Zirgaitis, Gretchen; Haischer, Michael H.; Beilfuss, Rachel; Uhrich, Toni D.; Hunter, Sandra K.; Wrucke, David |
| 14 | Prevalence of Face Mask Wearing in Northern Vermont in Response to the COVID-19Pandemic | Public Health Rep. | Beckage, Brian; Buckley, Thomas E.; Beckage, Maegan E. |
| 15 | Adherence to social distancing and wearing of masks within public transportation during the COVID-19 19 pandemic | Transportation Research Interdisciplinary Perspectives | Dzisi, E.K.J.; Dei, O.A. |
| 16 | Factors associated with incorrect facemask use among individuals visiting high-risk locations during COVID-19pandemic | Journal of Public Health and Development | Gunasekaran, S.S.; Gunasekaran, S.S.; Gunasekaran, G.H.; Zaimi, N.S.I.; Halim, N.A.A.; Halim, F.H.A. |
| 17 | Management and use of filter masks in the "none-medical" population during the COVID-19period | Saf. Sci. | Cumbo, Enzo; Scardina, Giuseppe Alessandro |
| 18 | Effectiveness of innovation media for improving physical distancing compliance during the COVID-19pandemic: A quasi-experiment in thailand | Int. J. Environ. Res. Public Health | Chutiphimon, Hattaya; Thipsunate, Apinya; Cherdchim, Atigun; Boonyaphak, Bootsarakam; Vithayasirikul, Panat; Choothong, Patiphan; Vichathai, Swit; Ngamchaliew, Pitchayanont; Vichitkunakorn, Polathep |
| 19 | The face mask-touching behavior during the COVID-19pandemic: Observational study of public transportation users in the greater Paris region: The French-mask-touch study. | J Transp Health | Guellich, Aziz; Tella, Emilie; Ariane, Molka; Grodner, Camille; Nguyen-Chi, Hoai-Nam; Mahe, Emmanuel |
| 20 | An Observational Study of Mask Guideline Compliance In An Outpatient OB/GYN Clinic Population | Eur. J. Obstet. Gynecol. Reprod. Biol. | Newman, Mark G. |
| 21 | Installation of pedal-operated alcohol gel dispensers with behavioral nudges and changes in hand hygiene behaviors during the COVID-19pandemic: A hospital-based quasi-experimental study | J. Public Health Res. | Wichaidit, Wit; Liabsuetrakul, Tippawan; Naknual, Sommanas; Kleangkert, Nanta |
| 22 | Mask use among pedestrians during the COVID-19pandemic in Southwest Iran: an observational study on 10,440 people | BMC Public Health | Rahimi, Zahra; Cheraghian, Bahman; Shirali, Gholam Abbas; Araban, Marzieh; Mohammadi, Mohammad Javad |
| 23 | Comparison of Face-Touching Behaviors before and during the Coronavirus Disease 2019 Pandemic | JAMA Netw. Open | Chen, Yong-Jian; Chen, Jie; Wu, Xiang-Yuan; Li, Xing; Qin, Gang; Xu, Jian-Liang; Feng, Ding-Yun |
| 24 | Compliance measurement and observed influencing factors of hand hygiene based on COVID-19guidelines in China | Am. J. Infect. Control | Zhou, Qian; Zhang, Xinping; Lai, Xiaoquan; Tan, Li |
| 25 | Adherence to personal protective equipment use among Healthcare workers caring for confirmed COVID-19and alleged non-COVID-19patients | Antimicrob. Resist. Infect. Control | Neuwirth, Meike M.; Mattner, Frauke; Otchwemah, Robin |
| 26 | COVID-19and face mask use: A st. kitts case study | Open Access Maced. J. Med. Sci. | Kungurova, Yulia; Brewster, Evelyn; Mera, Ritha; Ali, Khalil; Fakoya, Adegbenro O.J. |
| 27 | Facemask wearing to prevent COVID-19transmission and associated factors among taxi drivers in Dessie City and Kombolcha Town, Ethiopia | PLoS ONE | Natnael, Tarikuwa; Berihun, Gete; Abebe, Masresha; Adane, Metadel; Alemnew, Yeshiwork; Andualem, Atsedemariam; Ademe, Sewunet; Tegegne, Belachew |
| 28 | FACE COVERING ADHERENCE IN AN OUTPATIENT OPHTHALMOLOGY CLINIC DURING COVID-19-19 | Ophthalmic Epidemiol. | Parikh, Ankur; Kondapalli, Srinivas |
| 29 | Behavior in the use of face masks in the context of COVID-19-19 | Public Health Nurs. | Kellerer, Jan D.; Rohringer, Matthias; Deufert, Daniela |
| 30 | Community-based observational assessment of compliance by the public with COVID-1919 preventive measures in the south of Saudi Arabia. | Saudi J Biol Sci | Gosadi, Ibrahim M; Daghriri, Khaled A; Shugairi, Ahmad A; Alharbi, Ali H; Suwaydi, Abdullatif Z; Alharbi, Mohammed A; Majrashi, Ali A; Sumaily, Ibraheim A |
| 31 | Public Compliance with Face Mask Use in Honolulu and Regional Variation | Hawaii J Health Soc Welf | Tamamoto, Kasey A.; Rousslang, Nikki D.; Ahn, Hyeong Jun; Better, Heidi E.; Hong, Robert A. |
| 32 | Adherence of the General Public to Self-protection Guidelines During the COVID-19Pandemic | Disaster Med Public Health Prep | Jabbari, Parnian; Taraghikhah, Nazanin; Jabbari, Forouq; Ebrahimi, Saied; Rezaei, Nima |
| 33 | How do the general population behave with facemasks to prevent COVID-19in the community? A multi-site observational study | Antimicrob. Resist. Infect. Control | Haudebourg, Thomas; Blanckaert, Karine; Deschanvres, Colin; Boutoille, David; Peiffer-Smadja, Nathan; Lucet, Jean-Christophe; Birgand, Gabriel |
| 34 | First report on smoking and infection control behaviours at outdoor hotspots during the COVID-19pandemic: An unobtrusive observational study | Int. J. Environ. Res. Public Health | Sun, Yuying; Lam, Tai Hing; Chen, Jianjiu; Zhang, Xiaoyu; Ho, Sai Yin; Cheung, Yee Tak Derek; Wang, Man Ping; Wu, Yongda; Li, William H. C. |
| 35 | Youth Mask-Wearing and Social-Distancing Behavior at In-Person High School Graduations During the COVID-19 Pandemic | J. Adolesc. Health | Mueller, Anna S.; Beardall, Katherine A.; Millar, Krystina; Watkins, James T.; Diefendorf, Sarah; Abrutyn, Seth; O'Reilly, Lauren; Steinberg, Hillary |
| 36 | Towards Enforcing Social Distancing Regulations with Occlusion-Aware Crowd Detection | 16th IEEE International Conference on Control, Automation, Robotics and Vision, ICARCV 2020 | Cong, C.; Yang, Z.; Song, Y.; Pagnucco, M. |
| 37 | Obstetric Healthcare workers' adherence to hand hygiene recommendations during the COVID-19 pandemic: Observations and social-cognitive determinants. | Applied Psychology: Health and Well-Being | Derksen, Christina; Keller, Franziska M; Lippke, Sonia |
| 38 | Implications of a US study on infection prevention and control in community settings in the UK | British Journal of Community Nursing | Dowding, D.; McDonald, M.V.; Shang, J. |
| 39 | Achieving Perfect Hand Washing: an Audit Cycle with Surgical Internees | Indian Journal of Surgery | Mukherjee, R.; Roy, P.; Parik, M. |
| 40 | Observational study of compliance with infection control practices among Healthcare workers in subsidized and private residential care homes | BMC Infectious Diseases | Au, J.K.L.; Suen, L.K.P.; Lam, S.C. |
| 41 | Who Wears a Mask? Gender Differences in Risk Behaviours in the COVID-19Early Days in Taiwan | Economics Bulletin | Chuang, Y.; Chung-En Liu, J. |
| 42 | The impact of COVID-19pandemic on hand hygiene performance in hospitals | journal of infection control | Moore LD, Robbins G, Quinn J, Arbogast JW |
| 43 | The impact of coronavirus disease 2019 (COVID-19-19) on provider use of electronic hand hygiene monitoring technology | [Infect Control Hosp Epidemiol.](https://www.ncbi.nlm.nih.gov/pmc/articles/PMC7711352/) | Hess OCR, Armstrong-Novak JD, Doll M, et al |
| 44 | Surveillance of the infection prevention and control practices of healthcare workers by an infection control surveillance-working group and a team of infection control coordinators during the COVID-19pandemic | J Infect Public Health | Choi UY, Kwon YM, Kang HJ, et al. |
| 45 | Rigorous Hand Hygiene Practices Among Health Care Workers Reduce Hospital-Associated Infections During the COVID-19Pandemic. | J Prim Care Community Health. | Roshan R, Feroz AS, Rafique Z, Virani |
| 46 | Use of Proper Personal Protective Measures among Parents of Children Attending Outpatient Department - An Observational Study | Indian J Pediatr | Clinton M, Sankar J, Ramesh V, Madhusudan M. |
| 47 | Observation of Hand Hygiene Practices in Home Health Care. | J Am Med Dir Assoc. | McDonald MV, Brickner C, Russell D, et al. |
| 48 | Observing COVID-19related behaviours in a high visitor use area of Arches National Park | PLoS One. | Miller ZD, Freimund W, Dalenberg D, Vega M. |

**Table S4: NON-COVID-19 papers included in synthesis**

| Study | Title | Journal | Authors |
| --- | --- | --- | --- |
| 1 | Mentor's hand hygiene practices influence student's hand hygiene rates. | Am J Infect Control | Snow, Michelle; White, George L Jr; Alder, Stephen C; Stanford, Joseph B |
| 2 | Handwashing patterns in two dermatology clinics. | Dermatology | Cohen, H A; Kitai, E; Levy, I; Ben-Amitai, D |
| 3 | Infection control among professional tattooists in Minneapolis and St. Paul, MN. | Public Health Rep | Raymond, M J; Pirie, P L; Halcon, L L |
| 4 | Should self-assessment methods be used to measure compliance with handwashing recommendations? A study carried out in a French university hospital. | Am J Infect Control | Moret, Leila; Tequi, Brigitte; Lombrail, Pierre |
| 5 | A comparison of observed and self-reported compliance with universal precautions among emergency department personnel at a Minnesota public teaching hospital: Implications for assessing infection control programs | ANN. EMERG. MED. | Henry, K.; Campbell, S.; Maki, M. |
| 6 | Hand hygiene before donning nonsterile gloves: Healthcare workers' beliefs and practices. | Am J Infect Control | Baloh, Jure; Thom, Kerri A; Perencevich, Eli; Rock, Clare; Robinson, Gwen; Ward, Melissa; Herwaldt, Loreen; Reisinger, Heather Schacht |
| 7 | Is evidence guiding practice? Reported versus observed adherence to contact precautions: a pilot study. | Am J Infect Control | Jessee, Mary Ann; Mion, Lorraine C |
| 8 | Hand Hygiene Compliance Study at a Large Central Hospital in Vietnam. | Int J Environ Res Public Health | Le, Cam Dung; Lehman, Erik B; Nguyen, Thanh Huy; Craig, Timothy J |
| 9 | Do wearable alcohol-based handrub dispensers increase hand hygiene compliance? - a mixed-methods study. | Antimicrob. resist. infect. control | Keller, Jonas; Wolfensberger, Aline; Clack, Lauren; Kuster, Stefan P; Dunic, Mesida; Eis, Doris; Flammer, Yvonne; Keller, Dagmar I; Sax, Hugo |
| 10 | Beyond entry and exit: Hand hygiene at the bedside. | Am J Infect Control | Woodard, Jennifer A; Leekha, Surbhi; Jackson, Sarah S; Thom, Kerri A |
| 11 | Comparison of patient and healthcare professional perceptions of hand hygiene practices with the monthly internal audit at a tertiary medical center, Illinois 2010 | Am. J. Infect. Control | Soyemi, Caroline |
| 12 | Medical students and hospital hand hygiene - What do they know, and what do they do? | Surg. Infect. | Alemayehu, Hanna; Ho, Vanessa P.; Leviter, Julie I.; Drusin, Lewis M.; Barie, Philip S. |
| 13 | Effectiveness of a hand hygiene improvement program in doctors: Active monitoring and real-time feedback | Antimicrob. Resist. Infect. Control | Kim, S.R.; Cho, M.H.; Kim, W.J.; Song, J.Y.; Cheong, H.J. |
| 14 | Mind the mind: Results of a hand-hygiene research in a state-of-the-art cancer hospital | Indian J. Med. Microbiol. | Dalen, R.; Gombert, K.; Bhattacharya, S.; Datta, S. |
| 15 | A quasi-experimental study to determine the effects of a multifaceted educational intervention on hand hygiene compliance in a radiography unit | Antimicrob. Resist. Infect. Control | O'Donoghue, Margaret; Ng, Suk-Hing; Suen, Lorna K.P.; Boost, Maureen |
| 16 | Promoting Handwashing Behavior: The Effects of Large-scale Community and School-level Interventions | Health Econ. | Galiani, Sebastian; Gertler, Paul; Ajzenman, Nicolas; Orsola-Vidal, Alexandra |
| 17 | Knowledge and practice of infection control-in the NDM1 era | Antimicrob. Resist. Infect. Control | Lakshmi, V.; Ghafur, A.; Mageshkumar, K.; Karupusamy, C. |
| 18 | Flawed self-assessment in hand hygiene: A major contributor to infections in clinical practice? | Journal of Clinical Nursing | Kelcikova, Simona; Mazuchova, Lucia; Bielena, Lubica; Filova, Lenka |

***Table S5: NIH quality assessment checklist***

| Criteria | Yes | No | Other (CD, NR, NA)* |
| --- | --- | --- | --- |
| 1. Was the research question or objective in this paper clearly stated? |  |  |  |
| 2. Was the study population clearly specified and defined? |  |  |  |
| 3. Was the participation rate of eligible persons at least 50%? |  |  |  |
| 4. Were all the subjects selected or recruited from the same or similar populations (including the same time period)? Were inclusion and exclusion criteria for being in the study prespecified and applied uniformly to all participants? |  |  |  |
| 5. Was a sample size justification, power description, or variance and effect estimates provided? |  |  |  |
| 6. For the analyses in this paper, were the exposure(s) of interest measured prior to the outcome(s) being measured? |  |  |  |
| 7. Was the timeframe sufficient so that one could reasonably expect to see an association between exposure and outcome if it existed? |  |  |  |
| 8. For exposures that can vary in amount or level, did the study examine different levels of the exposure as related to the outcome (e.g., categories of exposure, or exposure measured as continuous variable)? |  |  |  |
| 9. Were the exposure measures (independent variables) clearly defined, valid, reliable, and implemented consistently across all study participants? |  |  |  |
| 10. Was the exposure(s) assessed more than once over time? |  |  |  |
| 11. Were the outcome measures (dependent variables) clearly defined, valid, reliable, and implemented consistently across all study participants? |  |  |  |
| 12. Were the outcome assessors blinded to the exposure status of participants? |  |  |  |
| 13. Was loss to follow-up after baseline 20% or less? |  |  |  |
| 14. Were key potential confounding variables measured and adjusted statistically for their impact on the relationship between exposure(s) and outcome(s)? |  |  |  |
